# Food Effects on Oral Drug Absorption: Application of Physiologically-Based Pharmacokinetic Modeling as a Predictive Tool

**DOI:** 10.3390/pharmaceutics12070672

**Published:** 2020-07-17

**Authors:** Lisa Cheng, Harvey Wong

**Affiliations:** Faculty of Pharmaceutical Sciences, The University of British Columbia, Vancouver, BC V6T 1Z3, Canada; lisa.cheng@alumni.ubc.ca

**Keywords:** food effects, physiologically-based pharmacokinetic model, oral absorption, mathematical modeling

## Abstract

The bioavailability of an orally administered small molecule is often dictated by drug-specific physicochemical characteristics and is influenced by many biological processes. For example, in fed or fasted conditions, the transit time within the gastrointestinal tract can vary, confounding the ability to predict the oral absorption. As such, the effects of food on the pharmacokinetics of compounds in the various biopharmaceutics classification system (BCS) classes need to be assessed. The consumption of food leads to physiological changes, including fluctuations in the gastric and intestinal pH, a delay in gastric emptying, an increased bile secretion, and an increased splanchnic and hepatic blood flow. Despite the significant impact of a drug’s absorption and dissolution, food effects have not been fully studied and are often overlooked. Physiologically-based pharmacokinetic (PBPK) models can be used to mechanistically simulate a compound’s pharmacokinetics under fed or fasted conditions, while integrating drug properties such as solubility and permeability. This review discusses the PBPK models published in the literature predicting the food effects, the models’ strengths and shortcomings, as well as future steps to mitigate the current knowledge gap. We observed gaps in knowledge which limits the ability of PBPK models to predict the negative food effects and food effects in the pediatric population. Overall, the further development of PBPK models to predict food effects will provide a mechanistic basis to understand a drug’s behavior in fed and fasted conditions, and will help enable the drug development process.

## 1. Introduction

Oral administration is the most common route of drug delivery because of the convenience, relative cost-effectiveness, and ease of administration. These combined factors promote patient compliance. Despite its many advantages, it is often difficult to predict the drug’s oral pharmacokinetic profile in vivo. Specifically, there exists intra- and interindividual variabilities in oral drug absorption which affects bioavailability and impacts the therapeutic outcome. Drug-specific physicochemical characteristics (i.e., solubility and permeability) dictate the drug’s ability to travel across or through the intestinal wall into the hepatic portal vein and offers an initial understanding of its oral pharmacokinetics. Furthermore, complex biological processes including fluctuations in pH, gastric and intestinal movement, rate of blood flow, and bile salt secretion can impact the orally administered drug’s rate of absorption.

The gastrointestinal tract’s physiology influences drug absorption and it is therefore imperative to ensure that any situations deterring the digestive system from homeostasis are explored. Food ingestion is known to alter the gastrointestinal environment, and individuals typically consume three meals a day. This could affect the frequency at which drugs can be administered and when they can be taken. According to the Food and Drug Administration (FDA), drug administration in the fasted condition occurs when there is an overnight fast lasting for a minimum of 10 h and no food is consumed until after at least 4 h of drug dosing [1]. In comparison, a fed state is achieved by following the same overnight fast, but patients are advised to take a meal within 30 min before administration with no eating until a minimum of 4 h afterward [1]. Concomitant food consumption and oral drug administration can affect drug absorption, thereby impacting its safety and efficacy. Increased systemic exposure can enhance efficacy, but also increases the probability of experiencing dose-related toxicities. Alternatively, an inefficient absorption decreases the bioavailability, potentially resulting in reduced efficacy or no efficacy at subtherapeutic levels. An ideal drug would not exhibit any significant meal-induced changes, allowing patients to adhere to their prescription without being concerned about their prandial state. However, this is not always possible, and patients need to be aware of any concerns that come with simultaneous food and drug administration.

**Figure 1:**
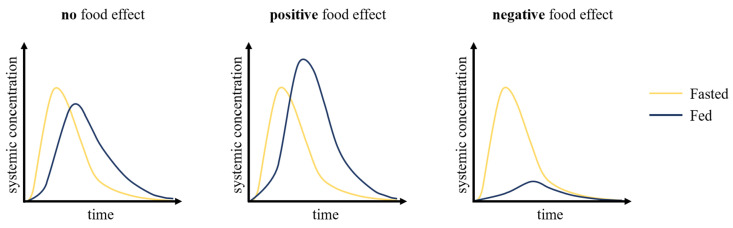
Drug pharmacokinetic profiles for the fasted and fed states. In the cases of no food effect, changes in the pharmacokinetic profile are minor and within the normal variations observed following oral administration. Positive food effects are associated with increases in systemic drug exposure and negative food effects are associated with decreases in drug exposure. Adapted from reference [3].

The food effects (Figure 1) on oral drug absorption can be investigated through preclinical and clinical pharmacokinetic studies, by determining whether oral therapeutics exhibit consequential or inconsequential effects when administered with food. Historically, the inability to accurately characterize a drug’s pharmacokinetics resulted in many drug development failures and, along with it, billions of dollars in losses. Traditionally, allometric scaling was used to translate the data obtained from the preclinical phase in drug development, which includes in vitro and in vivo studies, to first-in-human studies. However, this approach does not address the inter-species differences that are critical to consider to accurately predict drug pharmacokinetics, including transporters and drug-metabolizing enzymes [2]. Over the past few decades, there has been an evolution from non-compartmental modeling to complex physiologically-based pharmacokinetic (PBPK) models to ensure that the experimental drug will demonstrate safety and clinical efficacy. They offer many applications in pharmaceutical research throughout the many phases of drug development, from discovery to phase III clinical trials.

PBPK models aim to mimic human anatomy and physiology through a series of interconnected compartments, representative of physiological organs or tissues linked by systemic blood circulation. These mathematical models mechanistically predict drug pharmacokinetics (absorption, distribution, metabolism, and excretion) upon administration. Many commercially available modeling platforms can be used to build PBPK models, and some provide preset PBPK models while others allow the user to create their own models. All models have a set of assumptions that the user needs to be aware of, in order to understand the implications of a generated *a priori* simulation. Drug specific parameters, such as the molecular weight, log *p* (octanol:water partition coefficient), pKa, particle size, aqueous solubility, and metabolic intrinsic clearance, serve as inputs to the models. This information is obtained from in vitro and in vivo studies, or otherwise taken from the literature. Various conditions can be simulated by manipulating the input parameters. Plasma concentration–time curves can be generated, and relevant pharmacokinetic parameters, such as the rate of absorption and elimination, can be estimated. These results help guide medicinal chemists in optimizing the drug dosage formulation and provide dose regimen recommendations for the earlier and later phases of clinical trials. PBPK models are also used to mechanistically predict drug liabilities such as drug–drug interactions and food effects.

Their rapid development and incorporation into regulatory submissions to support clinical decisions call for a need to review the available models to inform the industry, academia, and regulatory agencies of their advantages and limitations. This review provides a general background on food effects and the use of PBPK models in predicting the food effects on oral drug absorption in humans.

## 2. Impacts of Food Consumption on Physiological Processes and Oral Drug Absorption

**Figure 2:**
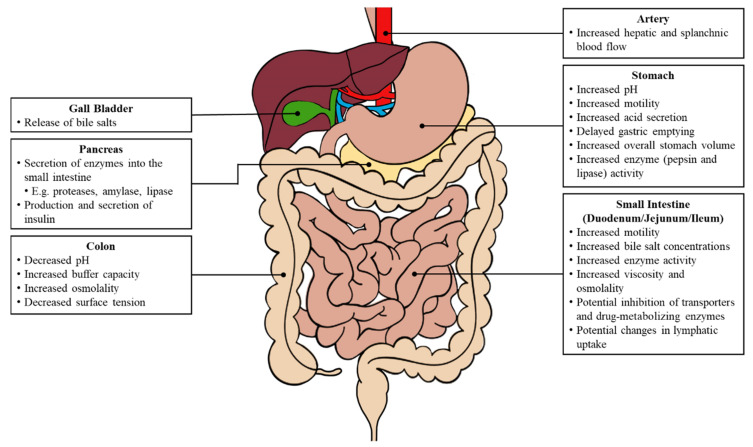
Major physiological changes in the gastrointestinal tract following food consumption. Adapted from reference [3].

The initiation of various biological processes is triggered by food consumption (Figure 2). Changes in both the gastric and intestinal system can impact oral drug absorption, including fluctuations in the gastrointestinal pH, an increased gastrointestinal motility, a delayed gastric emptying and prolonged intestinal transit, increased luminal fluids, the release of bile salts, and an increased hepatic and splanchnic blood flow [4].

Under fasted conditions, the stomach environment ranges from pH 1 to pH 3 in healthy individuals and holds 250 mL of luminal fluids [5,6]. The ingested food is chemically and mechanically digested into a viscous mixture, known as chyme. After food consumption, the gastric pH transiently increases from an averaged value of 1.88 to 4.98 and remains elevated for up to 4.5 h [5]. There is also an elevated gastric fluid volume of 500 mL or more, and the fluid dynamics are based on the caloric intake (low- or high-fat) of the meal. A more basic pH can alter the solubility and dissolution of ionizable compounds [6]. The proximal stomach relaxes to welcome food and generates a continuous tonic contraction, whereas the distal stomach produces phasic contractions that mix and break down the food. The gastric emptying time is longer and the process involving the migrating motor complex allows for the chyme to pass from the stomach, through the duodenum and subsequently enter the small intestine.

The pH levels in the duodenum and small intestine do not fluctuate as dramatically as the gastric pH, and remain around pH 6 to pH 8 [3]. The volume in the small intestine increases from 200 to 1000 mL [6]. The pH profile along the intestinal tract is dynamic under fed conditions and largely depends on several factors, including the composition of food ingested, gastric secretion, and bile salt concentrations. The release of bile salts by the gall bladder enhances the solubility of drugs with poor water-solubility. Furthermore, the intestinal motility in the post-prandial state exhibits different patterns based on the intraluminal solubility and viscosity [3]. An increase in the luminal viscosity reduces the water diffusivity, which consequently slows the disintegration of various dosage forms.

Drug absorption is also affected by changes in the luminal metabolism, food–drug interactions, and inhibition of transporters. The inhibition of the drug metabolism enzymes or efflux transporters within the gastrointestinal tract can allow for a greater percentage of drug absorption. For example, grapefruit juice is a well-known cytochrome P450 (CYP) 3A4 inhibitor and its co-administration with drugs metabolized by CYP3A4 can have significant impacts on the bioavailability [4]. Moreover, the lymphatic uptake also contributes to the absorption of drugs with large molecular weights or lipophilic compounds, and this process is increased following the consumption of high-fat meals [7]. Consequently, there is an increased drug absorption into the plasma compartment.

Finally, relative to the fasted state, a concomitant oral drug administration with food can severely impact the systemic bioavailability since changes in the gastrointestinal physiology influence the drug’s transit time, luminal dissolution, and permeability.

## 3. BCS and Regulatory Agencies’ Requirements for Food Effect Studies

The inception of the biopharmaceutics classification system (BCS) drug classes has revolutionized the regulation of therapeutics worldwide [8]. This scientific framework is used to classify drugs or active pharmaceutical ingredients into one of four classes based on their aqueous solubility and intestinal permeability (Table 1).

The BCS is recognized by various international regulatory agencies, including the Food and Drug Administration and European Medicines Agency. The system can help guide oral drug development as it triggers early assessments of food effects, but it cannot be used to quantitatively predict the magnitude of the changes in absorption. Factors that affect solubility and permeability include the pH-dependent solubility, interactions with contents in the gastrointestinal tract, and drug stability.

In 2005, Wu and Benet [9] created the Biopharmaceutics Drug Disposition Classification System (BDDCS) which uses the same criteria as the BCS for solubility but replaces the high and low permeability with extensive and poor metabolism, respectively. Their rationale behind this substitution was that the metabolism is comparably easier and less expensive to characterize than the intestinal permeability. To date, the FDA and European Medicines Agency (EMA) still refer to the BCS in their guidance documents [1,10].

Biowaiver extensions are granted to orally administered immediate-release BCS class I and III compounds, meaning that in vitro dissolution tests act as a surrogate for the pharmacokinetic data in lieu of the clinical food effect bioavailability and fed bioequivalence studies [1,10]. This class of drugs has a high fraction absorbed and they are typically not affected by food consumption; as such, class I drugs can be administered regardless of a fasted or fed state. Immediate-release BCS class II, III, and IV drugs, or modified-release products, are more likely to exhibit food effects and regulatory agencies mandate conducting a food effect bioavailability or a fed bioequivalence study.

Poorly soluble drugs are likely to exhibit a higher systemic exposure when administered with food. Increased bile salt concentrations promote lipophilic drug dissolution in the small intestine via the formation of micelles and some foods can inhibit the transporter activity in the intestinal mucosal cells [3]. On the contrary, negative food effects are commonly associated with drugs whose absorption is limited by their permeability. These effects are especially pronounced in drugs that have a narrow window of absorption, typically in the upper intestinal tract. Lastly, drugs with poor solubility and low permeability (BCS class IV drugs) pose significant problems for an effective oral administration. Their absorption does not follow any trends and depends on whether the rate-limiting step is dissolution or permeability. A list of orally administered drugs defined as essential therapeutics by the World Health Organization (WHO) has been classified into their respective BCS classes previously [11].

There is an abundance of novel small molecules that exhibit clinically relevant biological activity, and their physicochemical properties (e.g., lipophilicity) indicate a need to determine the food effects to avoid dosing in the subtherapeutic or supratherapeutic range. Relevant pharmacokinetic parameters, such as the total drug exposure (AUC_0-inf_), peak exposure (C_max_), time to peak exposure (T_max_), apparent clearance (Cl/F), and apparent volume of distribution (Vd/F) will be determined from the bioavailability studies to determine whether there is a positive, an absence of, or negative food effect. According to the FDA, a food effect is significant if “the 90% [confidence interval] for the ratio of population geometric means between fed to fasted treatments, based on log-transformed data, is not contained in the equivalence limits of 80–125% for AUC_0-inf_ (AUC_0-t_ when appropriate) or C_max_” [1].

## 4. Human Oral Absorption Models to Predict Food Effects

Physiologically-based absorption models are dynamic and they are used to predict oral drug absorption. These mechanistic models were preceded by the mixing tank model [12], which described the gastrointestinal tract as a single homogenous compartment. There were other multi-compartment models, ranging from two to four compartments [13,14], used to describe drug absorption through the intestinal tract, but they were also not reflective of the human small intestine. Common features among these models are equations describing the dissolution, permeation, and movement through the gastrointestinal tract, as these are fundamental processes related to oral absorption that are impacted by food co-administration. Dissolution of the solid oral drug formulation in the gastrointestinal fluid is described by the Noyes–Whitney equation [15]:

(1) $$\frac{dX_{sol}}{dt}=k_{diss}\times \left(S-\frac{X_{sol}}{V}\right)$$

where *k_diss_* represents the drug-specific dissolution constant, *S* is the drug solubility, and *X_sol_* refers to the amount of the drug in solution at time *t*, and *V* is the volume of the intestinal fluid. The dissolution rate constant and concentration gradient impact the release from the drug dosage form into the luminal fluids.

The drug can then permeate the gastrointestinal wall into the hepatic portal vein. Similar to dissolution, permeation follows first-order kinetics (Equations (2) and (3)). The rate at which the drug permeates the intestinal wall (*dX_perm_/dt*) is dependent on the compound-specific effective permeability (*P_eff_*), which is calculated based on an equation derived from Fick’s law of diffusion.

(2) $$\frac{dX_{perm}}{dt}=k_{a}\times X_{sol}$$

(3) $$k_{a}=\frac{2\times P_{eff}}{R}$$

where *k_a_* is the absorption rate constant, and *R* represents the small intestine’s radius.

In addition to dissolution and permeation, luminal metabolism and transporter activity can be incorporated into absorption models. Both factors exhibit nonlinear, saturable kinetics and their activities can be impacted following the consumption of certain foods. For example, the effects of grapefruit juice on drug-metabolizing enzymes, transporters, and P-glycoprotein are well established [16,17,18]. However, the changes in cytochrome P450 and transporter activity are not typically considered in physiologically-based absorption models when investigating the effects of food on bioavailability. The standard meal used in food effect studies, defined by the FDA and EMA, does not consist of foods known to significantly impact drug metabolism or transport.

The equations describing the fundamental processes of oral drug absorption are embedded into PBPK models, which are frequently used to investigate food effects on bioavailability. Lin et al. [19] provide a review of the fundamental processes integrated into PBPK models. Transit compartment models offer the advantage to divide the gastrointestinal tract into a series of compartments since it exhibits regional differences [20]. Examples of these transit compartment models include the compartmental absorption and transit (CAT), advanced CAT (ACAT), and advanced dissolution absorption metabolism (ADAM) model.

### 4.1. Compartmental Absorption and Transit Model

**Figure 3:**
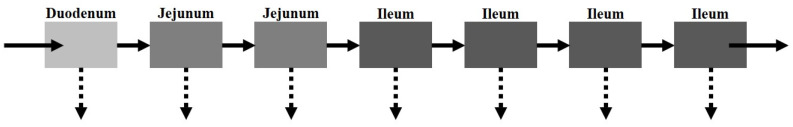
Schematic of the compartmental absorption and transit (CAT) model. The small intestine is separated into seven compartments. The solid and dotted arrows represent the transit rate constant (*k_t_*) and absorption rate constant (*k_a_*), respectively.

The CAT model, proposed by Yu et al. [21], separates the small intestinal tract into a series of seven transit compartments: duodenum, jejunum (×2), and ileum (×4 (Figure 3)). Each of the segments has the same residence time, but differs in volume and flow rate. The residence time varies depending on the absence or presence of food. Drug transit through the CAT model can be mathematically explained by a differential question (Equation (4)) that factors in the amount of the drug in a specific compartment (*M*), time (*t*), transit rate constant (*k_t_*), and the number of compartments (*N*) [18,21].

(4) $$\frac{dM_{n}}{dt}=k_{t}M_{n-1}-k_{t}M_{n}-k_{a}M_{n}$$

where n=1, 2, 3, …, N.

There are several model assumptions: (i) linear transfer kinetics from one compartment to the next, (ii) instantaneous mixing allowing for uniform concentrations, (iii) minimal absorption in the stomach or large intestine. Similar to the major disadvantage of the mixing tank model, the CAT model does not account for the pre-systemic (gut and hepatic) metabolism and cannot be used to explain the absorption of non-degradable drugs.

The inception of the CAT model established an introductory framework for others to advance models to mechanistically quantify oral drug absorption.

### 4.2. Advanced Compartmental Absorption and Transit Model

**Figure 4:**
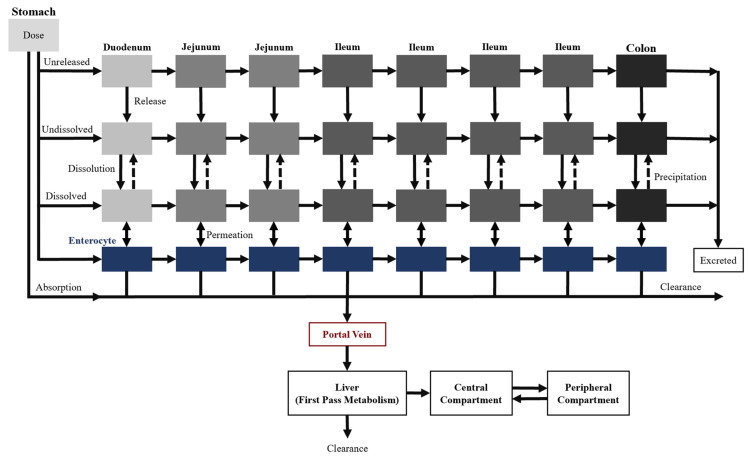
Schematic of the advanced compartmental absorption and transit (ACAT) model. Additional features (i.e., absorption in the stomach and colon compartment, clearance by first pass or gut metabolism, and drug release) were used to capture the mechanism of oral drug absorption.

Two additional transit compartments were added to the seven CAT model compartments to create the ACAT model, which is currently available on GastroPlus^®^, a commercially available software (Figure 4) [22]. The incorporation of the stomach and colon augment the predictive power of oral drug absorption by accounting for the pre-systemic metabolism and any colon absorption. Moreover, dissolution-limited absorption can be assessed using this model where the dissolution is described by a modified Noyes–Whitney equation [15]. The different states of the drug substance (e.g., unreleased, undissolved, and dissolved), drug-specific physicochemical characteristics (e.g., pKa and particle size), and gastrointestinal physiological conditions (e.g., gastric emptying time and pH) are taken into consideration as well. Overall, these input parameters allow the model to describe the mechanistic changes that impact oral drug absorption along the gastrointestinal tract.

### 4.3. Advanced Dissolution Absorption Metabolism Model

The ADAM model is also based on the CAT model and is implemented in the SimCYP^®^ software. The main difference between the ADAM and ACAT model is that the dissolution is calculated using the Wang–Flanagan generalized model [23] rather than the Noyes–Whitney equation.

The Grass model [24] is another oral absorption model that accounts for the drug permeability and solubility, and tissue surface area [18]. However, it does not consider drug degradation, pre-systemic metabolism, or the active transport of drugs. Overall, the most popular physiologically-based absorption models used to predict the effects of food are the ACAT and ADAM models.

PBPK modeling is used to guide the molecular design optimization and predict a safe and efficacious dose regimen for clinical trials based on in vitro, preclinical in vivo, and in silico pharmacokinetic data. The model is built with a limited amount of information during early discovery. As more data are collected from various studies and integrated into the model, it becomes more sophisticated. This reiterative process allows for model predictions to be performed with greater confidence and increases the probability of successful drug development.

Despite the efforts put into model optimization, there are currently no models that can perfectly capture oral drug absorption due to its complexity. As such, it is crucial to identify that there are limitations to the physiologically-based absorption models in its pure form alone. For example, there is no consideration of the degradation or active transport in the ADAM model [25]. The incorporation of oral drug absorption models into whole-body PBPK models combine drug ADME (absorption, distribution, metabolism, and excretion) within software platforms to mitigate the limitations of absorption-only models.

## 5. Predicting Food Effects

### 5.1. In Vitro Studies

The intestinal lumen environment has been recreated in vitro to assess the drug’s permeability and identify the potential mechanisms of the food effects. A monolayer of human colon epithelial cell lines (Caco-2 cells) mimics the intestinal mucosa in vitro and this model is useful in exploring a drug candidate’s transport from the intestinal lumen to systemic circulation [26]. Permeability studies were originally executed using a buffered transport solution (e.g., Hanks’ balanced salt solution) that maintains the ionic equilibrium and cell viability [27]. There are disadvantages to using a hydrophilic solution since most new chemical entities are lipophilic and poorly soluble in an aqueous medium, which can lead to a low recovery due to the intracellular accumulation or adsorption to the plastic material [28].

In the past couple of decades, there has been a shift towards using simulated intestinal fluids (SIFs) because of its resemblance to in vivo physiological conditions. Fasted state simulated intestinal fluids (FaSSIF) and fed state simulated intestinal fluids (FeSSIF) contain bile salts (e.g., taurocholate), phospholipids (e.g., phosphatidylcholine and lecithin), and/or fatty acids. Gastric fluids have also been simulated for the fasted (FaSSGF) and fed (FeSSGF) states. Over the years, the compositions of these simulated fluids have been optimized several times to more closely resemble the characteristics of the human in vivo environment, creating new ‘versions’ such as FaSSIF-V2 and FeSSIF-V2 [29,30,31]. The results from transport experiments using biorelevant, rather than compendial, media can better predict in vivo intestinal absorption [32].

Multiple in vitro studies were performed with FaSSGF, FeSSGF, FaSSIF, and FeSSIF to measure the initial dissolution rate and the overall dissolution profile. Transfer models using a scaled-down USP paddle apparatus are often the method of choice to assess a drug’s solubility and precipitation in both fed and fasted states [33]. This in vitro system aims to mimic the movement of dissolved and undissolved drug from the stomach into the small intestine. The dissolution of nelfinavir mesylate in FaSSIF-V2 and FeSSIF-V2 were quantified using the scaled-down paddle set up to evaluate the potential food effects in vivo. There was an 11-fold increase for the in vitro fed to fasted dissolution ratio, indicative of a positive food effect, but it was an overestimate of the observed food effects in healthy human volunteers (5-fold increase) [34]. It is hypothesized that the fasting stomach (FaSSGF) facilitates a dissolution faster than that observed in FaSSIF-V2, resulting in supersaturation and subsequently, drug precipitation in the fasted small intestine. In contrast, the solubility of nelfinavir in FeSSGF and FeSSIF-V2 were similar, indicating that no supersaturation nor drug precipitation is expected in the fed state [34]. These results illustrate that the significance of the stomach and small intestine’s contributions towards drug absorption cannot be fully captured with in vitro experiments.

Although in vitro studies provide useful information, there are limitations to their reliability in translation into humans. The transfer model does not describe the absorption of a drug from the lumen, impacting the equilibrium that dictates the drug precipitation which is particularly significant when predicting the food effect of BCS class II weak bases [35]. Despite the described limitations, they serve as important inputs for PBPK models that characterize changes in the in vivo pharmacokinetic profiles of drugs under fasted and fed conditions.

### 5.2. In Vivo Studies

Food effect studies are commonly conducted in the canine model since they can be fed a solid formulation with food composition akin to the human diet, and their luminal characteristics show similarities with humans. Nevertheless, there is a lack of confidence in using canine models because they often overpredict the food effects in humans [36,37]. Species-specific anatomical features and physiological processes present challenges in extrapolating in vivo data to clinical trials. For example, the small and large intestines are comparably shorter in the canine model and the fasting gastric pH of beagle dogs is reported as higher than humans [36]. The use of pentagastrin has been shown to decrease the gastric pH so that the study results reflect the expected outcomes in humans [38]. Other models that may be used to study food effects include pigs, rats, and mice, and a detailed comparison of the digestive system is outlined in a review by Sjören et al. [36].

Mathematical models can be used to describe the anatomy and physiology of the animal model and it has been previously suggested that they provide more accurate forecasts than allometric scaling [2]. An adaptation of the ACAT model incorporating species-specific parameters was successful in predicting the food effects for theophylline (BCS class I drug) in both dogs and humans [39]. However, the mathematical model could not correctly simulate a plasma concentration–time curve for aprepitant, a poorly soluble compound. There was a significant difference in the predicted and observed data of fasted dogs and this was attributed to a miscalculation of the regional solubility, leading to an overprediction of the solubility enhancing food effects [36,39]. Overall, the importance of preclinical in vivo studies investigating food effects is that they can serve as a means to validate PBPK models describing food effects in animals. This validation in animals would occur prior to the translation of these PBPK models to humans, so that prospective predictions of food effects in humans can be performed [40].

### 5.3. PBPK Models Predicting Food Effects in Humans

Some of the first mathematical models used to assess the effects of the concomitant intake of food on pharmacokinetics were one- [41], two- [42], and three-compartment [43] distribution models. However, food as a categorical covariate in these models inadequately described the physiological changes in the gastrointestinal system in response to food consumption. A one-compartment open model of sustained-release fenofibrate absorption was developed from clinical data and a plasma concentration–time curve was previously simulated [41]. A delay in the T_max_, as a result of slower gastric emptying rates, was observed after food consumption. In a subsequent study, a more complex mechanism-based pharmacokinetic model with four physiological and one central compartment was developed to evaluate the changes in the gastrointestinal system from the pre- to post-prandial state, and the ensuing fenofibrate absorption [44]. The stomach and duodenum volumes were fixed for the fasting and fed conditions, and all other pharmacokinetic parameters, including the gastric emptying rate constant to the duodenum compartment, duration of the food effect, and effect of the gastric emptying rate and bile acid on absorption, were fitted based on the clinical data. Although both the one-compartment and the mechanism-based pharmacokinetic model characterized positive changes in the C_max_ and AUC, the latter could mechanistically quantify the effects of each physiological parameter in the fasted and fed (standard and high-fat meals) states. PBPK models serve as an expansion of mechanism-based pharmacokinetic models and provide an even deeper understanding of specific mechanisms influencing the food effect and oral drug absorption in general.

Numerous biological processes are initiated following food consumption, as outlined in the earlier section, and these physiological changes can be captured in mechanistic PBPK models by adjusting the model parameters. Commercially available software such as GastroPlus^®^ and SimCYP^®^ have often been used to evaluate the food effects on compounds from all BCS classes. These softwares have subtle differences in the physiological parameters used to describe the prandial states including the gastric emptying time, gastric volume, and bile salt concentrations throughout the gastrointestinal tract [45]. Since GastroPlus^®^ and SimCYP^®^ provide the encoded ACAT and ADAM models, many values describing the physiological differences between the fasted and fed states are not reported in published literature since these parameter values may not be easily accessible. In contrast, there exist platforms that allow researchers to custom-build PBPK models with compartments representative of the gastrointestinal tract linked by differential equations that describe important oral absorption processes.

#### 5.3.1. Changes in the Physiological Parameters in PBPK Models in Fasted and Fed States

Various changes in the physiological parameters in PBPK models that influence drug absorption following food consumption have been identified. Specifically, the gastric emptying time, gastric pH, and increase in bile salt concentrations throughout the intestinal tract are physiological processes that can enhance drug solubility and are important to consider when describing the food effects for poorly soluble compounds [18]. These parameters are common adjustments made to PBPK models to capture food effects and appear to be sufficient in capturing the food effects on many BCS class II compounds including ketoconazole [35], posaconazole [35], and celecoxib [46]. Despite the successful prediction of food effects, the understanding of the oral absorption of drugs, in general, is still a challenge. For the three examples of BCS class II compounds listed, the optimization of the volume of distribution terms, using observed plasma concentration–time data, was required for the assembly of their PBPK models prior to the simulations of food effect. Further, for ketoconazole and posaconazole, the in vivo solubility conditions estimated using the observed intraluminal concentration profiles were required for the construction of their respective PBPK models. Finally, a majority of the published PBPK models describe BCS class II drugs that exhibit positive food effects [45]. These models provide little advancements in understanding the implications of food consumption on BCS class III and IV compounds.

#### 5.3.2. In Focus: PBPK Models for Low Solubility, Low Permeability Compounds

The physiochemical characteristics of BCS class IV compounds significantly impact rate-limiting processes governing oral absorption, and thereby present the greatest obstacle when predicting food effects. Typically, positive food effects are correlated with drugs displaying a solubility-limited absorption, whereas it is largely hypothesized that low permeability compounds can exhibit a decrease in bioavailability under fed conditions [6]. Since the dissolution, solubility, and permeability are all limiting factors for BCS class IV compounds, there is no visible trend of food effects in this class. Understanding the adjustments made to PBPK models for class IV compounds under fed conditions will inform on the parameters that need to be focused on when predicting food effects on class II and III compounds since class IV compounds exhibit both their absorption rate-limiting properties. Literature reports of PBPK models used to predict the food effects in class IV compounds have been compiled and the adjustments made in these models can be separated into physiological and compound-specific parameters (Table 2). The gastric emptying time, gastric pH, gastrointestinal volume, and bile salt concentrations are physiological parameters that were consistently taken into account. In terms of the compound-specific parameters, the compound’s solubility under human in vivo fasted vs. fed conditions, or the biorelevant solubility, was a common modification in the PBPK models. It is important to highlight that these same adjustments are made in PBPK predictions of food effects for compounds from other BCS classes as well. Finally, for some of the compounds in Table 2, the post-absorptive distribution parameters (including the apparent volume of distribution) used in the PBPK models were obtained from fitting the plasma concentration–time profiles from food effect studies to a one- or two-compartment model.

The food effects on BCS class IV compounds have been predicted using PBPK models with some success. For example, the predicted vs. observed clinical pharmacokinetic parameters of venetoclax given with a low-fat meal was within the 0.8- to 1.25-fold range criteria, indicating that the model could accurately predict the observed food effects [47]. However, it must be noted that it was necessary to optimize the permeability of venetoclax in order to account for an underprediction of the plasma concentration–time profile in the fasted state prior to the food effect predictions. Despite the success of the food effect prediction for a low-fat meal, the simulated venetoclax plasma concentration–time profile associated with the co-administration of a high-fat meal severely underpredicted drug exposure when compared with clinical data. A limitation of the PBPK model for venetoclax is its inability to capture the change in lymphatic uptake under fed conditions [47]. Dietary lipids, especially from a high-fat meal, can enhance the absorption of lipophilic drugs (log *p* > 5) significantly through an increased lymphatic uptake [6]. Lymphatic absorption plays an important role in the oral absorption of venetoclax which has a log *p* of 8. Predictions of the observed positive food effect of a high-fat meal on venetoclax likely require the incorporation of the lymphatic uptake process into the mechanistic PBPK model [47].

The food effects on a second compound, NVS113, were not accurately predicted using PBPK models. The human PBPK model predicted an insignificant food effect, whereas clinical data showed an approximate 30% decrease in the AUC following food consumption [40]. Interestingly, NVS113 is the only BCS class IV compound in Table 2 with negative food effects. The authors attributed the overprediction to potential food–drug interactions or transporter interactions [40].

#### 5.3.3. Lack of Predictability for Drugs with Negative Food Effects

A majority of the compounds that have been studied for food effects fall under the BCS class II category [45]. There is a wealth of information related to characterizing positive food effects which predominately captures the physiological changes that enhance drug solubility. In contrast, the representation of PBPK models that predict negative food effects is lacking. The number of PBPK models that fail to predict the pharmacokinetic profile of BCS class II compounds with negative food effects further highlights the insufficient understanding of the mechanisms that contribute to a decrease in bioavailability.

The predictive power of mathematical models is a direct translation of the information inputted into the model. There is no clear consensus on which parameters hold the greatest significance when predicting food effects. The majority of physiological parameters (see Table 2) that have been adjusted in PBPK models have been shown to be predictive of positive food effects; however, they are likely insufficient to predict negative food effects. One parameter that has been thought to profoundly influence a decreased bioavailability is the viscosity of the gastrointestinal fluid [52]. Meal-induced viscosity prevents water penetration into a tablet, thus impeding its disintegration and dissolution. When the release of a drug is delayed, it can only be absorbed at a downstream site resulting in a significant decrease in drug bioavailability. Zolpidem, a BCS class I drug, surprisingly exhibits negative food effects. In an investigation of the observed food effect of zolpidem, in vitro data from biorelevant dissolution testing and permeability assays were incorporated into the ACAT and ADAM models to inform a mechanistic understanding of zolpidem absorption [53]. The simulated plasma concentration–time profiles illustrated a decreased C_max_ and increased T_max_ value, corresponding with a decreased bioavailability following food consumption [53,54]. However, both models underpredicted the negative food effect on modified-release tablets in the fed and fasted state. Drug absorption appeared to be largely influenced by the gastric emptying time and viscosity of gastric media. A subsequent study investigating the influence of food on zolpidem absorption when administered as an immediate release tablet using an in vitro—in silico approach confirmed that a higher viscosity of chyme led to an underestimation of the negative food effects [55]. These results suggest that gastrointestinal fluid viscosity may be an important parameter to incorporate into PBPK models that can aid in the prediction of negative food effects.

In addition to viscosity, potential food–drug interactions, the induction of pre-systemic metabolism, drug degradation, and inhibition of transporters need to be further investigated to determine whether these phenomena need to be accounted for when modeling negative food effects [18,40]. Food–drug interactions can limit the concentration of the drug that is available for absorption, which may contribute to the decreased bioavailability of drugs exhibiting low permeability. This was previously mentioned in a prospective clinical study examining the differences in the changes of the pharmacokinetics of an experimental BCS class IV compound, NVS113, in the fed vs. fasted state, and this hypothesis was revisited when discussing a second compound, NVS001. A retrospective analysis of a clinical study investigating the experimental drug NVS001 revealed that the C_max_ and AUC of this BCS class III drug decreases significantly following food consumption. Heimbach et al. [40] applied a scaling factor to account for the inhibition of the uptake transporter OATP2B1 by food, and concluded that their model with this adjustment could accurately predict the pharmacokinetic profile of NVS001 under fed conditions.

#### 5.3.4. Overall Performance of PBPK Models Describing Food Effects in Humans

A majority of the PBPK models published in the literature simply indicate that their simulations are “predictive” or “not predictive”, without including their criteria. PBPK model performance is typically evaluated through goodness-of-fit plots and visual predictive checks. Recently, Li et al. [45] assessed the predictive performance of the PBPK models used to characterize the food effects on oral absorption and reported that among the 48 food effect simulated cases, almost 50% (23 of 48) were successful in forecasting the observed food effects within a 25% bounder (1.25-fold). When the boundary was increased to 100% (2-fold), 75% (36 of 48) of the models were able to predict the observed food effects [45]. These results suggest that, with a more stringent success criteria, only a limited number of models were truly predictive of the food effects observed in humans and additional research needs to be conducted to inform the model outcome.

### 5.4. PBPK in the Pediatric Population

Relative to the adult population, there are a limited number of studies investigating the food effects on oral drug absorption in the pediatric population. Oftentimes, the outcomes observed in adults are scaled according to body surface area to make dosing recommendations for neonates, infants, and children. Food effects in the pediatric population cannot be fully disregarded when no clinically significant outcomes are observed in adults [56]. Current methods should be accepted with caution since neonates, infants, and children are not simply smaller adults. Additional considerations, including the different feeding patterns and gastrointestinal processes, must be addressed to fully capture changes in drug absorption.

The food composition and frequency of consumption vary significantly among the pediatric population and between the adult population. Neonates and young infants are likely fed milk as their primary source of nutrients, whereas there is a shift towards carbohydrate consumption in children [56]. Gastric emptying time is largely influenced by food composition rather than age [57]. For example, neonates that are fed a regular formula will have a greater gastric emptying time than if they were given an extensively hydrolyzed formula or breast milk [58]. There are innate differences in gastric emptying between neonates, infants, and adults, where infants start to reach adult values after 6 months of age [59]. Before that age, they exhibit a slower gastric emptying following food consumption which consequently increases the T_max_. Neonates and infants also consume meals at a greater frequency and are rarely truly fasted compared to adult standards [60]. Food acts as a buffer in the stomach and induces gastric acid secretion which impacts the dissolution of weakly acid and basic drugs. Infants have a higher postprandial gastric pH in comparison to adults and, like gastric emptying, the meal content is also a dependent factor [61]. Moreover, it is not uncommon for an infant or a child to receive an oral drug with a food or beverage (e.g., applesauce, yogurt, or fruit juice) to promote compliance.

There remain challenges in designing efficient processes to adequately assess the risk of food effects in the pediatric population. Clinical trials with neonates, infants, and children exploring the impact of food on drug absorption are not always feasible. Furthermore, there is an added complexity with the rapid developmental changes in both physiology and biochemistry of the gastrointestinal tract, resulting in a large variability in parameters cited in literature when describing the pediatric population. Here, PBPK models can be leveraged to predict the C_max_, T_max_, and AUC, and identify the potential consequences of dosing in a fed and fasted state. These models are typically created to predict pharmacokinetics in the adult population and then adapted to perform pediatric simulations [62,63]. Specific anatomical and physiological parameters must be adjusted accordingly to reflect the younger population.

Previously, Johnson et al. [60] integrated available pediatric gastrointestinal physiology into the ADAM model to predict the pediatric exposure and dosage of theophylline, paracetamol, and ketoconazole. Various gastrointestinal physiological processes, including the intestinal length and diameters, gastric emptying, and gastric pH, were adjusted to achieve a pediatric ADAM model that simulated concentration–time profiles, reflective of observed data for all three drugs. In infants prescribed with ketoconazole, there was a shorter T_max_ when administered with a liquid feed in comparison to the semi-solid/solid feed. This observation further supports that solid foods are associated with a slower gastric emptying than aqueous solutions. Furthermore, the authors suggest that a better understanding of the fluid volume dynamics in the gastrointestinal tract would help explain the lower fed to fasted fraction absorbed ratio of ketoconazole observed in the pediatric age group when compared to adults [60]. This study marks the first time a mechanistic absorption model was used to predict oral drug absorption for both fed and fasted states in the pediatric population.

A shift away from allometric power models to PBPK models to describe food effects in the pediatric population will be an asset to drug development. This will minimize off-label prescription which often leads to a suboptimal efficacy. To build confidence in these models, there needs to be a greater understanding of the gastrointestinal tract’s physiology and biochemistry in each developmental stage. Additionally, barriers to obtaining pediatric pharmacokinetic data need to be overcome for these models to be validated and further optimized. The reiterative optimization of pediatric PBPK models will be proven useful in guiding dose recommendations for all BCS classes while accounting for the prandial state in neonates and infants.

## 6. Current Limitations and Future Perspectives

Food effect studies inform drug product labeling to include specific instructions regarding food or beverage consumption with drug administration. The characterization of food effects is particularly important for drugs with indications in the critically ill and special populations. Reliance on positive food effects to achieve efficacious exposures for a BCS class II and IV cancer drug may not be ideal for those individuals who have difficulty consuming high-fat meals, which can discourage patient compliance [47,64]. In contrast, the negative food effects on certain drugs may not be ideal for specific populations since a drug dosing schedule will need to be followed. If food effects pose a challenge to attaining the optimal efficacy and the drug cannot be further optimized to mitigate these effects, a different route of administration should be considered.

Despite the level of predictability shown in Table 2, there is a low level of confidence in the predictions provided by PBPK models for drug absorption and food effects implying that there remain limitations that need to be addressed [65]. It is imperative to recognize that publication biases towards successful results are common, thus creating an illusion that the majority of PBPK models are predictive. In vitro and in vivo experiments have been adapted over the past few decades in order to resemble dissolution and precipitation in humans. Despite these efforts, these studies often do not yield data that reflect observed in vivo or clinical behavior. Food consumption induces many complex biological changes in the digestive system and the PBPK models published in the literature use a wide range of values to describe system parameters. Additional studies are needed in order to fully understand the phenomena that govern the extent of food effects.

Moreover, some physiological changes following food consumption are not well characterized, such as lymphatic uptake. Under fed conditions, there is an increased lymphatic uptake of the drug, thereby increasing the concentration in systemic circulation. A PBPK model used to describe venetoclax absorption following a high-fat meal was not predictive, and the researchers suggested that lymphatic uptake influences drug absorption to a greater degree than what was previously hypothesized [47]. The example of venetoclax, a lipophilic compound, illustrates how high-fat meals, or increased caloric intake, pose a challenge in uncovering the food effects.

Additionally, there remain many unknowns that have yet to be fully characterized. There is a lack of knowledge on how bile–micelle binding, and enzymatic and transporter activity contribute to food effects that needs to be addressed in future studies. Although food has been shown to inhibit various intestinal drug-metabolizing enzymes and transporters [66], this information is seldom incorporated into mathematical models. Different food compositions can impact the activity of enzymes and transporters, and the degree to which they positively or negatively influence drug absorption varies. A major challenge in bridging this gap is the heterogeneity within the population since an individual’s diet largely influences the diversification of the gastrointestinal tract.

It is also crucial to note that a majority of the food effect studies are explored in adults. In the pediatric population who undergo rapid development changes, physiological processes following food consumption amplify the complexity of characterizing drug absorption. In special populations, there is also sparse data collection, which poses a challenge to validate a PBPK model. Here, sensitivity analyses are useful to determine the value of drug parameters if there is missing or unreliable data.

PBPK models are relatively inexpensive and valuable tools used to guide the course of drug development. Despite their advantages, there are many improvements that must be made in order to build confidence in the model’s outputs. The current understanding of biological processes following food consumption limits the accuracy and validity of PBPK models and, consequently, any conclusions drawn from PBPK models need to be accepted with caution.

## 7. Conclusions

In this review, the application of PBPK models to predict food effects on drug absorption was explored. Various complex physiological processes are initiated upon food consumption, which can enhance or reduce a drug’s dissolution, solubility, and permeability. BCS classes act as a guide to determine whether a food effect exists, although additional studies need to be performed to capture the extent of food effects. In the past few decades, there have been strides in predicting meal-induced changes in pharmacokinetic parameters using PBPK models through reiterative optimization. Although there have been significant strides in understanding food effects, there remains a gap in the knowledge of biological processes following food consumption that needs to be bridged in order to accurately predict food effects on drug absorption. It is hopeful that these mathematical models can streamline the development of novel therapeutic compounds, starting with food effect predictions.

## Figures and Tables

**Table 1:** Properties and examples of drugs in the four biopharmaceutics classification system classes.

BCS Class	Properties	Examples
I	High solubility, high permeability	Propranolol, theophylline, zidovudine
II	Low solubility, high permeability	Carbamazepine, celecoxib, ketoconazole
III	High solubility, low permeability	Cimetidine, trospium, atenolol
IV	Low solubility, low permeability	Nelfinavir, venetoclax, furosemide

**Table 2:** Physiological and compound-specific parameters that have been adjusted in PBPK models to predict the food effects on borderline class II/IV and IV drugs.

Compound	BCS Class	Formulation	Food Effect	Meal	Platform	Predictive (Yes/No)	Physiological Parameters						Compound-Specific Parameter	
							Gastric Emptying	Gastric pH	Duodenum/Jejunum pH	Gastric Volume	Intestinal Fluid Volume	Bile Salt Concentration	Biorelevant Solubility	Ref.
Nelfinavir **	IV	Tablet	Increase	Standard Breakfast	STELLA^®^	Yes	X	X (using BM *)	NA	X	X	X (using BM *)	X	[34]
NVS113	IV	Solid Formulation	Decrease	High-fat	GastroPlus^®^	No	X ^1^	X ^1^	X^1^	X ^1^		X ^1^	X	[40]
Venetoclax	IV	Amorphous solid dispersions	Increase	Low-fat	SimCYP^®^	Yes	X ^1^	X ^1^	Duodenum only	X ^1^		X ^1^	X	[47]
Venetoclax	IV	Amorphous solid dispersions	Increase	High-fat	SimCYP^®^	No	X ^1^	X ^1^	Duodenum only	X ^1^		X ^1^	X	[47]
Aprepitant **	IV	Micronized/Nanosized	Increase	Standard Breakfast	STELLA^®^	Yes	X	X (using BM *)	NA	X	X	X (using BM *)	X	[48]
Cinnarizine **	II/IV	Tablet	Increase	Standard Breakfast (High-fat)	STELLA^®^	Yes	X	X (using BM*)	NA	X	X	X (using BM *)	X	[49]
Compound A **	IV	Tablet	Increase	Standard Breakfast	STELLA^®^	Yes	X	X (using BM *)	NA	X	X	X (using BM *)	X	[50]
Compound X	II/IV	Capsule	Increase	High-fat	GastroPlus^®^	Yes	X	X	X	X ^1^		X ^1^	X	[51]

* BM = Biorelevant media; ^1^ based on data available from reference [45]. ** PBPK models use post absorptive distribution parameters including the apparent volume of distribution from fitting plasma concentration–time profiles from food effect studies to a one- or two-compartment model.
